# Wernicke's Encephalopathy Mimicking Acute Onset Stroke Diagnosed by CT Perfusion

**DOI:** 10.1155/2014/673230

**Published:** 2014-02-23

**Authors:** Alok Bhan, Rajiv Advani, Kathinka D. Kurz, Elisabeth Farbu, Martin W. Kurz

**Affiliations:** ^1^Department of Neurology, Stavanger University Hospital, 4068 Stavanger, Norway; ^2^Neuroscience Research Group, Stavanger University Hospital, 4068 Stavanger, Norway; ^3^Department of Radiology, Stavanger University Hospital, 4068 Stavanger, Norway

## Abstract

*Background*. Metabolic syndromes such as Wernicke's encephalopathy may present with a sudden neurological deficit, thus mimicking acute onset stroke. Due to current emphasis on rapid admission and treatment of acute stroke patients, there is a significant risk that these stroke mimics may end up being treated with thrombolysis. Rigorous clinical and radiological skills are necessary to correctly identify such metabolic stroke mimics, in order to avoid doing any harm to these patients due to the unnecessary use of thrombolysis. *Patient*. A 51-year-old Caucasian male was admitted to our hospital with suspicion of an acute stroke due to sudden onset dysarthria and unilateral facial nerve paresis. Clinical examination revealed confusion and dysconjugate gaze. Computed tomography (CT) including a CT perfusion (CTP) scan revealed bilateral thalamic hyperperfusion. The use of both clinical and radiological findings led to correctly diagnosing Wernicke's encephalopathy. *Conclusion*. The application of CTP as a standard diagnostic tool in acute stroke patients can improve the detection of stroke mimics caused by metabolic syndromes as shown in our case report.

## 1. Case Report

A fifty-one-year old Caucasian male was admitted to our hospital with sudden onset confusion, dysarthria, and a unilateral facial palsy.

The patient was in his usual health prior to admission. His medical history consisted of hypothyroidism, peptic ulcer disease, and previous alcoholism complicated by alcoholic polyneuropathy. Home nursing services noticed an acute onset dysarthria and a unilateral facial nerve paresis so they contacted the emergency medical services. Due to the acute deterioration of his speech, he was admitted as a possible candidate for thrombolytic treatment-flown into the hospital by air ambulance.

**Figure 1:**
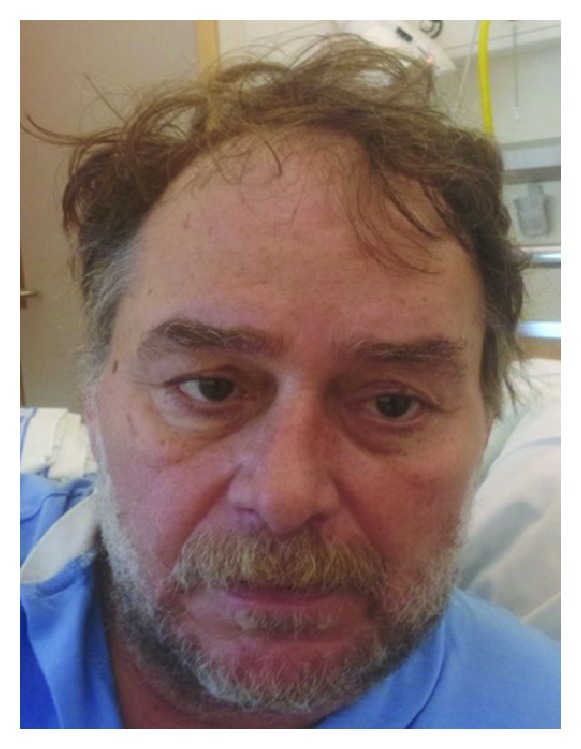
The patient with dysconjugate eye position.

Upon admission the patient was fully conscious but was perceived to be somewhat perplexed. He was oriented for neither date nor time. On examination, pupils were equal and reactive with corneal reflexes present bilaterally. Both visual fields were intact. Yet, his gaze in the midline was dysconjugate with abduction weakness present bilaterally (Figure 1). This was manifested clinically with double vision on lateral gaze. No nystagmus was observed. There was no facial asymmetry and on clinical testing no facial nerve paresis was noted. The tongue was not deviated from the midline with no appreciable atrophy or fasciculations. Examination of strength was consistent with a generalized weakness but there were no formal pareses. No involuntary movements were seen. Deep tendon reflexes were normal (2+) with bilateral flexor plantar response. The patient was able to localize tactile and noxious stimuli on both sides without any obvious side-to-side difference. His blood pressure was 170/110 mmHg, his ECG showed a normal sinus rhythm without any PQ segment or QRS complex pathologies, and there was however some T wave inversion in the inferior and lateral leads.

**Figure 2:**
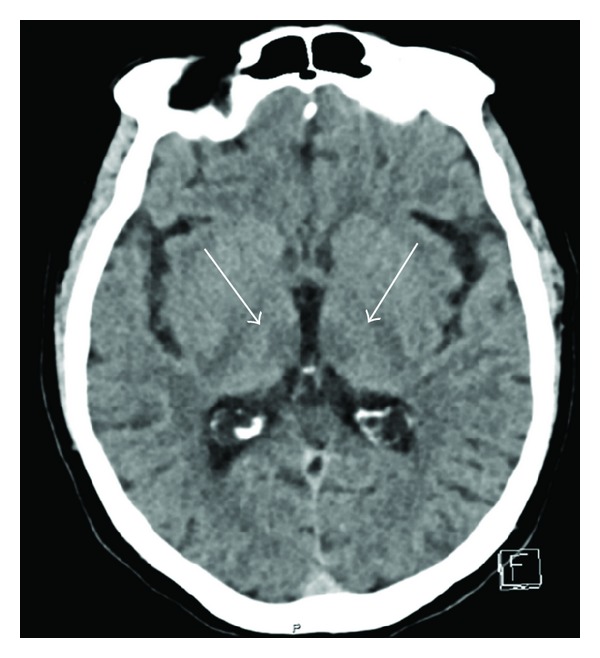
CT on admission. The white arrows show the hypodensities seen medial in the thalamus bilaterally.

**Figure 3:**
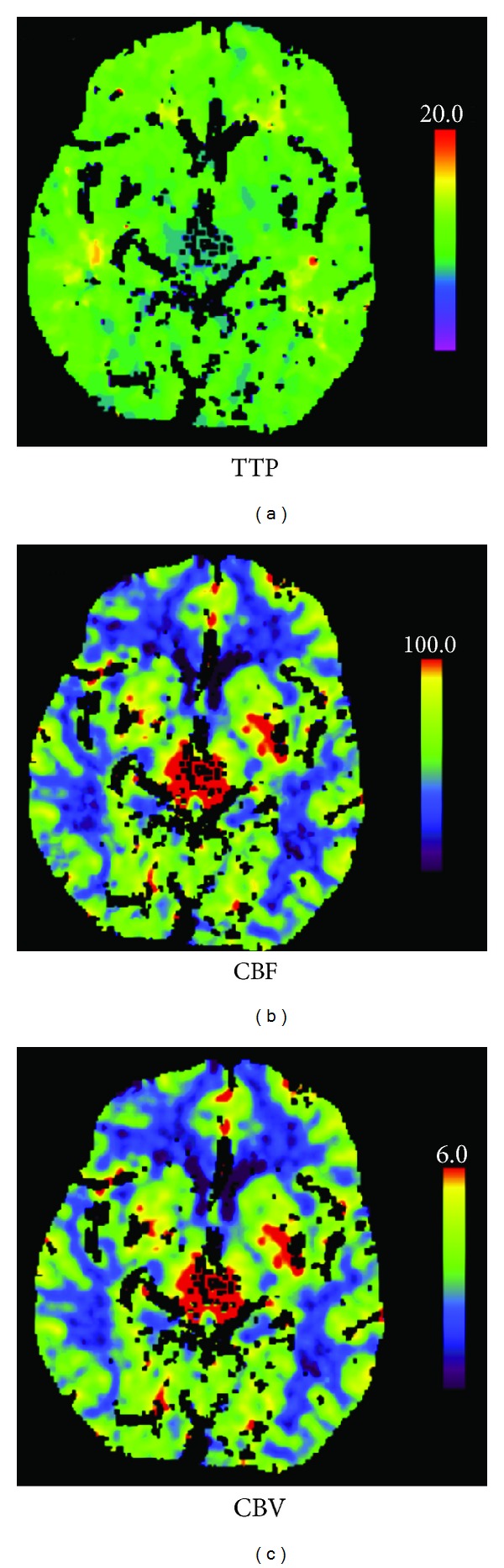
CT perfusion on admission. Time-to-peak map (TTP, (a)) showing reduced time-to- peak and cerebral blood flow map (CBF, (b)) and cerebral blood volume map (CBV, (c)) showing increased relative cerebral blood flow and relative cerebral blood volume in thalamus bilaterally.

Due to the acute onset of dysarthria and oculomotor pathologies an urgent cerebral CT was performed revealing hypodense changes in the thalamus bilaterally (Figure 2). A subsequent CT perfusion scan (CTP) revealed hyperperfusion in the same areas (Figure 3) whereas CT angiography of the posterior circulation vessels did not show any abnormalities.

A complete blood count was noteworthy for a slightly elevated glucose 7.9 mmol/L (reference range 4.0–6.0 mmol/L) and C-reactive protein 14 mg/L (reference range 0–7 mg/L). The remainder of the blood work-up, which included electrolytes, and kidney- and liver-function tests, was normal. Testing for intoxicants in the blood, which included opiates, benzodiazepines, and ethanol, was negative. A lumbar puncture was performed and analysis of the cerebrospinal fluid (CSF) was normal, showing a CSF protein of 0.45 g/L (reference range 0.0–0.5 g/L), 2 white blood cells per microliter (reference range 0–5 cells per microliter), and CSF glucose of 5 mmol/L. A chest X-ray was performed, revealing no pathologies. A transthoracic echocardiogram was also performed with normal findings.

**Figure 4:**
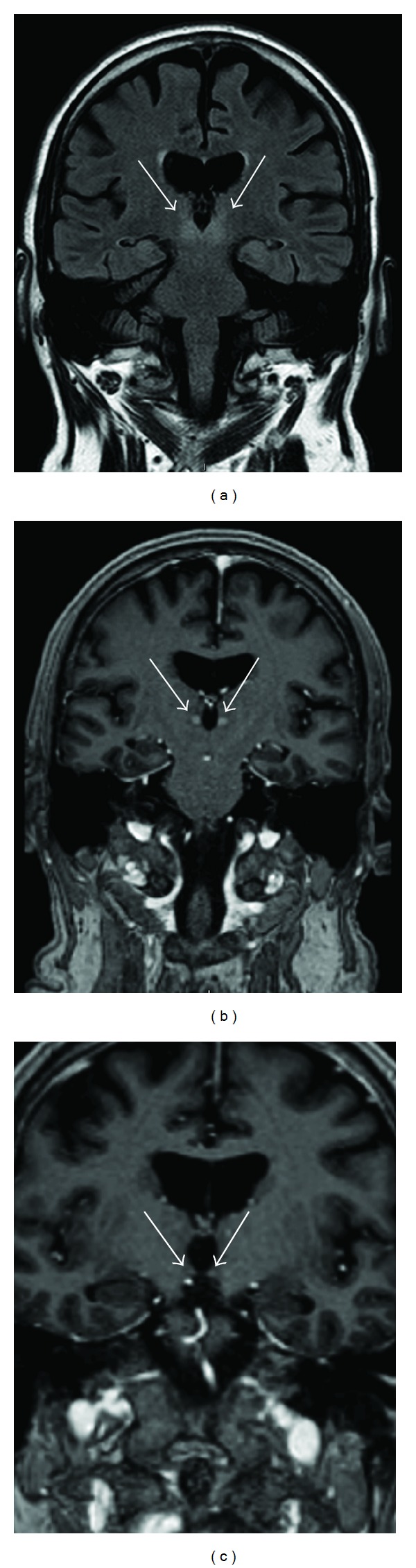
MR at day 3 showing hyperintense changes in both thalami adjacent to the third ventricle on FLAIR series (a) and Gd-enhancement in the thalami (b) and in the mammillary bodies (c).

MRI imaging of the brain showed bilateral hyperdense lesions in the thalamus on the fluid attenuated inversion recovery (FLAIR) series. These lesions exhibited slight gadolinium (Gd) contrast enhancement as did both mammillary bodies (Figure 4). The MRI showed no pathologies on the diffusion weighted images and the intracranial vessels were normal as visualized by the time of flight (TOF) technique.

Both the clinical presentation and the radiological examinations were consistent with Wernicke's encephalopathy. In addition, caregivers of the patient were contacted and confirmed that he had not been eating normally with increasing bouts of nausea and vomiting over a two- week period. Furthermore they informed us that he had been treated for Wernicke's encephalopathy several years ago, at another hospital. The patient was promptly treated with intravenous thiamine for three days. After starting treatment he showed significant clinical improvement on a daily basis. Vitamin B1 treatment was continued orally and the patient was discharged on the fourth day after admission, clinically completely restituted.

## 2. Discussion

Wernicke's encephalopathy is a relatively common, acute onset neuropsychiatric syndrome, arising from vitamin B1 (thiamine) deficiency; it is characterized by ophthalmoplegia, cerebellar dysfunction, and mental changes. Yet, this classical triad of symptoms is seen in only 16% of patients [1]. Although clinical studies predict a modest incidence of around 0.04–0.13%, post studies show a much higher incidence of about 0.8–2.8% [2], meaning that the vast majority of these cases remain undiagnosed.

The thiamine deficiency seen in Wernicke's encephalopathy leads to brain lesions usually restricted to more certain vulnerable areas. These lesions usually establish themselves within 2-3 weeks, corresponding well with the time needed for the depletion of the bodies thiamine reserves [3]. Thiamine is converted to thiamine pyrophosphate, fundamental for several biochemical pathways in the brain: ATP synthesis (carbohydrate metabolism), production and maintenance of myelin (lipid metabolism), and amino acids synthesis (protein metabolism) and in addition acetylcholinergic and serotoninergic synaptic transmission is also thiamine dependent [4, 5]. Thiamine deficiency and the resulting loss of osmotic gradient across cell membranes lead to cytotoxic edema, an initially reversible “biochemical lesion” which later transforms into an irreversible, structural lesion with the potential for permanent neurological deficit or death [6]. In our patient we strongly suspect that malnutrition led to his thiamine deficiency. The patient's caregivers reported that he was more or less bedridden a few weeks prior to admission resulting in serious subsequent underfeeding.

An acute onset stroke was suspected in our patient according to the FAST criteria (Face, Arm, Speech, and Time); the presence of a facial paresis and slurred speech, noted prehospitally, led to rapid admission [7]. As time is critical in the treatment of stroke patients, our patient, exhibiting oculomotor changes and dysarthria in the emergency room, was taken directly to the CT laboratory to exclude radiological contraindications for thrombolysis and to verify a possible stroke by CTP imaging. The unenhanced CT scan revealed bilateral hypodense changes in the thalamus (Figure 2). However, CT angiography showed no pathology in the vertebral and basilar arteries. CTP revealed, in stark contrast to our expectations, a hyperperfusion in the pertinent areas (Figure 3). This hyperperfusion in both thalami in addition to the typical clinical features (confusion and oculomotor pareses) led to the consideration of Wernicke's encephalopathy as a differential diagnosis. This diagnosis was confirmed by subsequent MRI images, showing hyperintense thalami on FLAIR series and Gd-enhancement in the thalami and in the mammillary bodies.

To our knowledge this is the first case report, where a patient admitted to hospital with the suspicion of an acute onset stroke was correctly diagnosed with Wernicke's encephalopathy, due to demonstrable hyperperfusion on CTP series. Interestingly, the unenhanced CT showed bilateral hypodensities in the thalamus, making basilar artery thrombosis a possible differential diagnosis. In the vast majority of Wernicke's encephalopathy cases, unenhanced CT is generally interpreted to be normal in the acute stages of the disease [8]. In subacute and chronic stages, unenhanced CT may indicate areas of variable hypoattenuation [9] and in extremely rare cases hemorrhagic lesions have been noted [10]. The gold standard in neuroradiologic diagnostics is MRI, demonstrating symmetrical high signal intensity alterations on T2 weighted images in the thalami, mammillary bodies, tectal plate, and periaqueductal area [8]. However, the absence of these neuroradiological findings does not exclude Wernicke's encephalopathy [11].

Due to the increased public awareness of possible stroke symptoms and the emphasis on rapid treatment administration in acute stroke patients, there is an increasing risk of treating stroke mimics with thrombolysis. Studies have shown that up to 30% of patients presenting with acute neurological deficits, considered to be acute onset stroke, have in fact stroke mimics [12]. Although thrombolysis seems to be relatively safe in stroke mimics [7, 13], rigorous clinical and radiological skills are the key in order to avoid doing any harm to these patients by limiting the use of unnecessary thrombolysis. In addition, patients presenting with stroke mimics could potentially need acute medical intervention other than thrombolysis, in order to avoid neuronal damage. We hypothesize that the application of CTP as a standard investigation in acute stroke patients would considerably improve the detection of stroke mimics caused by metabolic syndromes, as presented in our case report.
